# Effect of cadmium on young plants of *Virola surinamensis*

**DOI:** 10.1093/aobpla/plz022

**Published:** 2019-04-05

**Authors:** Waldemar Viana Andrade Júnior, Cândido Ferreira de Oliveira Neto, Benedito Gomes dos Santos Filho, Cristine Bastos do Amarante, Eniel David Cruz, Ricardo Shigueru Okumura, Antônio Vinícius Correa Barbosa, Diana Jhulia Palheta de Sousa, Jéssica Suellen Silva Teixeira, Anderson de Santana Botelho

**Affiliations:** 1 Institute of Agronomists Sciences, Laboratory of Biodiversity Studies of Upper Plants, Federal Rural University of the Amazon, Campus Belém, Pará, Brazil; 2 Museu Paraense Emílio Goeldi (MPEG), Belém, Pará, Brazil; 3 Pará/Institute of Exact and Natural Sciences, Federal University of Pará, Belém, Pará, Brazil; 4 Brazilian Agricultural Research Corporation (Embrapa), Belem, Pará, Brazil; 5 Institute of Agronomists Sciences, Federal Rural University of the Amazon, Campus Parauapebas, Pará, Brazil; 6 Institute ICIBE, Federal Rural University of the Amazon Campus Belém, Pará, Brazil

**Keywords:** Bioconcentration factor, photosystem II, phytostabilization

## Abstract

The steady increase in cadmium (Cd) levels in the environment from anthropogenic actions has contributed to environmental degradation. *Virola surinamensis* is a forest species that has desirable characteristics such as deep and dense roots, relatively rapid growth and high biomass production to remedy contaminated environments by Cd. The aim of this study was to assess the physiological responses and the phytoextraction and tolerance capacity of young plants of *V. surinamensis* submitted to Cd concentrations. The experimental design was a completely randomized design with five Cd concentrations (0, 15, 30, 45 and 60 mg L^−1^) for 60 days. Leaf water potential (Ψ_pd_), stomatal conductance (*gs*) and transpiration (*E*) reduced in plants exposed to Cd. Lower values of maximum photochemical efficiency of photosystem II (*Fv*/*Fm*), electron transport rate (*ETR*) and photochemical quenching coefficient (*qP*) were accompanied by reduction of photosynthesis (*A*) with increasing concentrations of Cd, although the non-photochemical quenching coefficient (*NPQ*), and intercellular CO_2_ concentration (*Ci*) showed increase. Instantaneous water-use efficiency (*A*/*E*), net photosynthesis to intercellular CO_2_ concentration ratio (*A/Ci*) and total chlorophyll (Chl) reduced with increasing levels of Cd. Cadmium concentrations increased in different plant tissues (root > stem > leaf). The tolerance index (TI) indicated that *V. surinamensis* presented medium and high tolerance to Cd. The results of bioconcentration factor (BCF) and translocation factor (TF) showed low plant efficacy in Cd phytoextraction and suggest that *V. surinamensis* may be promising for phytostabilization of Cd.

## Teaser

Identification of native Amazonian tree species with potential for phytoextraction and tolerance to cadmium (Cd) can be used to direct studies and phytoremediation programs for preservation of natural areas and restoration of environments contaminated by heavy metals. The study evaluated the physiological responses and the phytoextraction and tolerance capacity of young plants of Virola surinamensis to Cd. We founded that Virola surinamensis showed medium and high tolerance to Cd and low efficacy in Cd phytoextraction and suggest that Virola surinamensis may be promising for the phytostabilization of Cd.

## Introduction

The constant increase in cadmium (Cd) levels in the environment from agricultural and industrial activities has contributed to the degradation and contamination of soils, surface water and groundwater (Ahmad *et al.* 2014). This has created a major worldwide concern, especially as it is a non-biodegradable and easily absorbed, translocated and accumulated element in plant tissues (Ali *et al.* 2013), making it highly bioavailable and therefore toxic even at relatively low concentrations (Bashir *et al.* 2015).

The symptoms of phytotoxicity by Cd in plants include a modification in the indices of chlorophyll *a*, *b* and total, resulting in significant reductions in photosynthetic activity (Elloumi *et al.* 2014; Hernández *et al.* 2015; Yang *et al.* 2015; Michel-López *et al.* 2016; Zouari *et al.* 2016; Nikolić *et al.* 2017; Silva *et al.* 2017), especially due to the inhibition of photosystem II (PSII) (Di Baccio *et al.* 2014) by changing the potential yield of the photochemical reaction (*Fv/Fm*) (Fernández *et al.* 2013; Yang *et al.* 2015; Solti *et al.* 2016) and CO_2_-fixing key enzymes, such as ribulose-1,5-bisphosphate carboxylase (RuBisCO) (Tran and Popova 2013). In addition, Cd in plants affects the water relations, respiration (Oláh *et al.* 2015), transpiration, stomatal conductance and intercellular CO_2_ concentration (Elloumi *et al.* 2014; Song *et al.* 2016; Zouari *et al.* 2016; Nikolić *et al.* 2017).

In the Amazon, flooded ecosystems are constantly susceptible to contamination, as they are receptors for nutrients and organic and inorganic contaminants, including heavy metals (Khan *et al.* 2017). High concentrations of Cd in water and sediments of these areas were demonstrated in studies by Seyler and Boaventura (2003) and Oliveira *et al.* (2017). Among heavy metals, Cd is considered one of the most toxic. Thus, the demand for solutions to recover soils and aquifers contaminated by metals, among them the Cd (Zhao *et al.* 2015).

Phytoextraction is a promising phytoremediation technique and consists of the absorption of soil or water contaminants by the plant root and its translocation to shoot (Sharma *et al.* 2016). The success of this technique involving forest species on Cd removal depends on the higher accumulation capacity of the metal, high biomass production and plant tolerance (Nikolić *et al.* 2017). However, only some plants suitable for phytoextraction of Cd are hyperaccumulating, that is, they have superior capacity to extract, accumulate and tolerate high levels of the metal (Fan *et al.* 2011). These plants can accumulate >100 mg Cd kg^−1^ (dry weight) in the aerial part (Van der Ent et al. 2013).

Studies involving woody species native to the Amazon for phytoremediation of Cd are scarce. In the present study, *Swietenia macrophylla* (Fan *et al.* 2011) and *Cassia alata* (Silva *et al.* 2017) demonstrated a capacity to accumulate and tolerate Cd, while *Calophyllum brasiliense* (Pereira *et al*. 2017) presented compromised growth, with low accumulation and greater sensitivity to Cd. To identify tree species with a capacity for phytoextraction of Cd, they can serve to direct studies and programmes on phytoremediation for the preservation of natural areas and the recomposition of environments contaminated by these metals.

In this work, we consider *Virola surinamensis* (Ucuúba) as a forest species with a deep and dense root system, relatively rapid growth and high biomass production. These characteristics are considered desirable and effective for woody plants to remediate metal contaminated soils, such as Cd (Abdul Qados 2015). In addition, Ucuúba is widely distributed in Amazonian floodplain and igapó ecosystems, which are potentially subject to the presence of Cd. In addition, this species has been successfully used in reclamation programmes for degraded areas, including high concentrations of copper (Cu) and zinc (Zn) in the litter (Costa *et al*. 2017). Suggesting that *V. surinamensis* develops mechanism of tolerance to environments contaminated by heavy metals.

Considering that Cd tolerance is modulated by defence mechanisms and that no studies on the behaviour of *V. surinamensis* exposed to Cd have been found, we tested the hypothesis that young plants of *V. surinamensis* trigger different physiological strategies to tolerate environments contaminated by Cd. Thus, this study aimed to assess (i) the water potential, gas exchange and the fluorescence of chlorophyll *a* and (ii) Cd concentration in different plant organs, bioaccumulation, translocation, and the phytoextraction and tolerance capacity of young plants of *V. surinamensis* submitted to Cd concentrations.

## Methods

### Experimental site

The experiment was conducted in a greenhouse at the Federal Rural University of Amazonia (UFRA) in Belém, State of Pará, Brazil (01°27′21″S, 48°30′16″W), from 15 September 2017 to 14 November 2017. According to the climatic classification of Köppen, the climate is type Af (Tropical rainforest), with an annual average precipitation of 2921.7 mm, average temperature of 25.9 °C, average relative humidity of 86.8 % and wind speed of 1.35 m s^−1^ (Ramos *et al.* 2009).

### Plant material and growth condition

Seeds of *V. surinamensis* were collected in the area of the Brazilian Agricultural Research Corporation (Embrapa Eastern Amazon), located in Belém, State of Pará, Brazil (01°26′44.2″S, 48°25′03.8″W). These seeds were sown in 5-L polyethylene trays containing sand and sterilized sawdust (1:1, v/v), and maintained under mean air temperature (*T*_air_) and relative air humidity (RH) of 28 °C and 90 %. After emergence, the seedlings containing the first pair of eophylls were transplanted to 10-L polyethylene pots containing yellow latosol and poultry litter (3:1, v/v). The seedlings grown were in a greenhouse for 180 days, being irrigated daily to replace the water lost by evapotranspiration.

Subsequently, the young plants were removed and their roots washed with deionized water and transferred to 5-L Leonard pots containing sterilized and washed sand and 800 mL of nutrient solution of Sarruge (1975), replaced weekly and constituted of (µM): KH_2_PO_4_, 400; KNO_3_, 2000; Ca(NO_3_)_2_·4H_2_O, 2000; MgSO_4_·7H_2_O, 800; FeEDTA, 400; H_3_BO_3_, 400; MnCl_2_·4H_2_O, 400; ZnCl_2_, 400; CuCl_2_·2H_2_O, 400; and H_2_MoO_4_·H_2_O, 400. The pH was maintained at 5.9 ± 0.2 using HCl and NaOH. The ionic strength was initiated in 25 % (10 days) and then increased to 50 % (35 days), remaining for a period of acclimatization of 45 days.

### Experimental design and treatment evaluation

After 45 days of cultivation, we selected the most uniform seedling considering height, stem diameter, number of leaves and submitted to five Cd concentrations (treatments) as following: 0 mg L^−1^ of CdCl_2_ (control), 15, 30, 45 and 60 mg L^−1^ of CdCl_2_. The doses of Cd were determined based on the Resolution 420 of the National Council of the Environment, CONAMA (Brasil 2009), which establishes criteria and guiding values of soil quality regarding the presence of chemical substances. The experimental design was a completely randomized design with seven replications, per each treatment, totalling 35 experimental units. A single plant per pot was considered a replicate. All variables for treatment comparisons were assessed 60 days after Cd treatment differentiation.

### Leaf water potential, leaf gas exchange and total chlorophyll

Leaf water potential (Ψ_pd_) was determined in the morning between 0430 and 0530 h, using the Scholander’s pressure bomb (m 670, PMS Instrument Co., Albany, OR, USA), as described by Pinheiro *et al.* (2008). The third leaf from apices was used as sample.

The variables net CO_2_ assimilation rate (*A*), stomatal conductance to water vapour (*gs*), transpiration (*E*), intercellular CO_2_ concentration (*Ci*), ratio of the net photosynthesis and intercellular CO_2_ concentration (*A/Ci*) and instantaneous water-use efficiency (WUE, calculated as the ratio between *A* and *E*) were assessed using a portable infrared gas analyzer (LI-6400XT, LI-COR Biosciences Inc., Lincon, NE, USA) equipped with a blue/red light source (LI-6400-02B, LI-COR) under a photosynthetically active radiation (PAR) flux of 1000 µmol m^−2^ s^−1^ and CO_2_ flux of 400 ppm (Silvestre *et al.* 2017). The assessments of gas exchanges were carried out between 0900 and 1100 h, representing the daytime period in which photosynthesis reaches the maximum values, as determined from the diurnal curves of leaf gas exchanges. The measurements were always performed in completely expanded single sheets, located in the third node counted from the apex.

The total chlorophyll content (Chl) was determined using a portable chlorophyll meter (SPAD 502-plus, Konica Minolta, Osaka, Japan), with readings taken on the third adult leaf counted from the apex at three points on each side of the midrib of the adaxial leaf face (Jesus and Marrenco 2008). The results were expressed in SPAD (Soil Plant Analysis Development) index.

### Fluorescence of chlorophyll *a*

The fluorescence of chlorophyll *a* was determined on the third adult leaf, counted from the apex, using the LI-6400XT (LI-COR Biosciences Inc., Lincon, NE, USA). Leaves adapted to the dark for 30 min were illuminated with a weak pulse of modulated radiation to obtain the initial fluorescence (*F0*). A saturating white light pulse of 6.000 µmol m^−2^ s^−1^ was applied for 0.8 s to ensure maximum fluorescence emission (*Fm*). In the dark-adapted samples, the maximum photochemical efficiency of PSII was estimated by the ratio between variable and maximum fluorescence [*Fv/Fm* = (*Fm* − *F0*)/*Fm*]. Saturating white light pulses were applied to achieve the maximum fluorescence (*F′m*). The actinic light was then switched off and the far-red radiation switched on to measure *F0* adapted to the light (*F′0*). The capture efficiency of excitation energy by open PSII reaction centres (*F′v*/*F′m*) was estimated as the ratio (*F′m* − *F′0*)/*F′m*. The photochemical quenching coefficient (*qP*) was calculated as *qP* = (*F′m* − *Fs*)/(*F′m* − *F′0*) and the non-photochemical quenching coefficient (*NPQ*) was determined from the equation of Stern–Volmer [*NPQ* = (*Fm*/*F′m*) − 1] (Krause and Weis 1991). The actual quantum yield of PSII electron transport (ΦFSII) was calculated as (*Fm′* − *Fs*)/*Fm′* (Genty *et al.* 1989), where *Fs* is the steady state fluorescence. Electron transport rate (*ETR*) was calculated as *ETR* = *ΦPSII* × *PPFD* × *f* × *α*, where *PPFD* is the photosynthetic photon flux density, *f* is a factor that contributes to energy partitioning between PSII and PSI and is assumed to be 0.5, indicating that the excitation energy is equally distributed between the two photosystems, and *α* is the leaf absorbency by the photosynthetic tissues and is assumed to be 0.84 (Maxwell and Johnson 2000).

### Cadmium analysis

Cadmium analysis was processed in triplicate according to the methodology described by Miyazawa *et al.* (2009), with adaptations. The dry matter (0.5 g) of each sample was digested in a digester tube with 8 mL of nitric acid solution (HNO_3_) + perchloric acid (HClO_4_) (3:1). After cooling, the solution in the tube was filtered and diluted with deionized water to a final volume of 50 mL. Cadmium contents were determined in this solution by atomic absorption spectrometry (Thermo Scientific ICE 3000).

### Tolerance index

The tolerance index (TI) was determined to assess the plant ability to develop in the presence of Cd. The TI for Cd concentrations and for each plant organ was calculated according to Wilkins (1957), in which TI values can range from 0 (maximum sensitivity) to 1 (maximum tolerance).

$$\begin{array}{l} TI=\frac{DMP_{solution\;with\;Cd}{\;}_{\left(mg\right)}}{DMP_{control\;\;solution}{\;}_{\left(mg\right)}}\;\;\times \;100 \end{array}$$ (1)

where DMP solution with Cd is the dry mass of the plant in the solution with Cd and DMP control solution is the dry mass of the plant in the control solution.

### Bioconcentration and translocation factor

To assess Cd phytoextraction capacity in *V. surinamensis*, the bioconcentration (BCF) and translocation factor (TF) were calculated at the end of the experiment, as in Fan *et al.* (2011).

$$\begin{array}{l} BCF=\frac{C_{plant}}{C_{solution}} \end{array}$$ (2)

where *C*_plant_ is the sum of the concentration of Cd (mg kg^−1^) in the plant organs (root, stem and leaves) and *C*_solution_ is the metal concentration of the nutrient solution (mg L^−1^).

$$\begin{array}{l} TF=\frac{C_{aerial\;part}}{C_{root}} \end{array}$$ (3)

where *C*_aerial part_ is the sum of the concentration of Cd (mg kg^−1^) in plant organs (stem and leaves) and *C*_root_ is the concentration of the metal in the root of the plant (mg kg^−1^).

### Data analysis

The experimental data were assessed for the normality and homogeneity of variances by the Shapiro–Wilk and Bartlett tests, respectively. For parametric variables, the means of treatments were submitted to PROC GLM, *post hoc* Tukey’s HSD test and correlation between variables by the PROC CORR linear of Pearson using the software SAS 9.1.3 (SAS 2007). For non-parametric variables, the data were assessed by the Kruskal–Wallis test with Bonferroni correction by the software RStudio version 1.1.383. The experimental data of all analyses were assessed at 5 % significance.

## Results

### Effect of Cd on water potential, gas exchange and total chlorophyll

**Figure 1.**
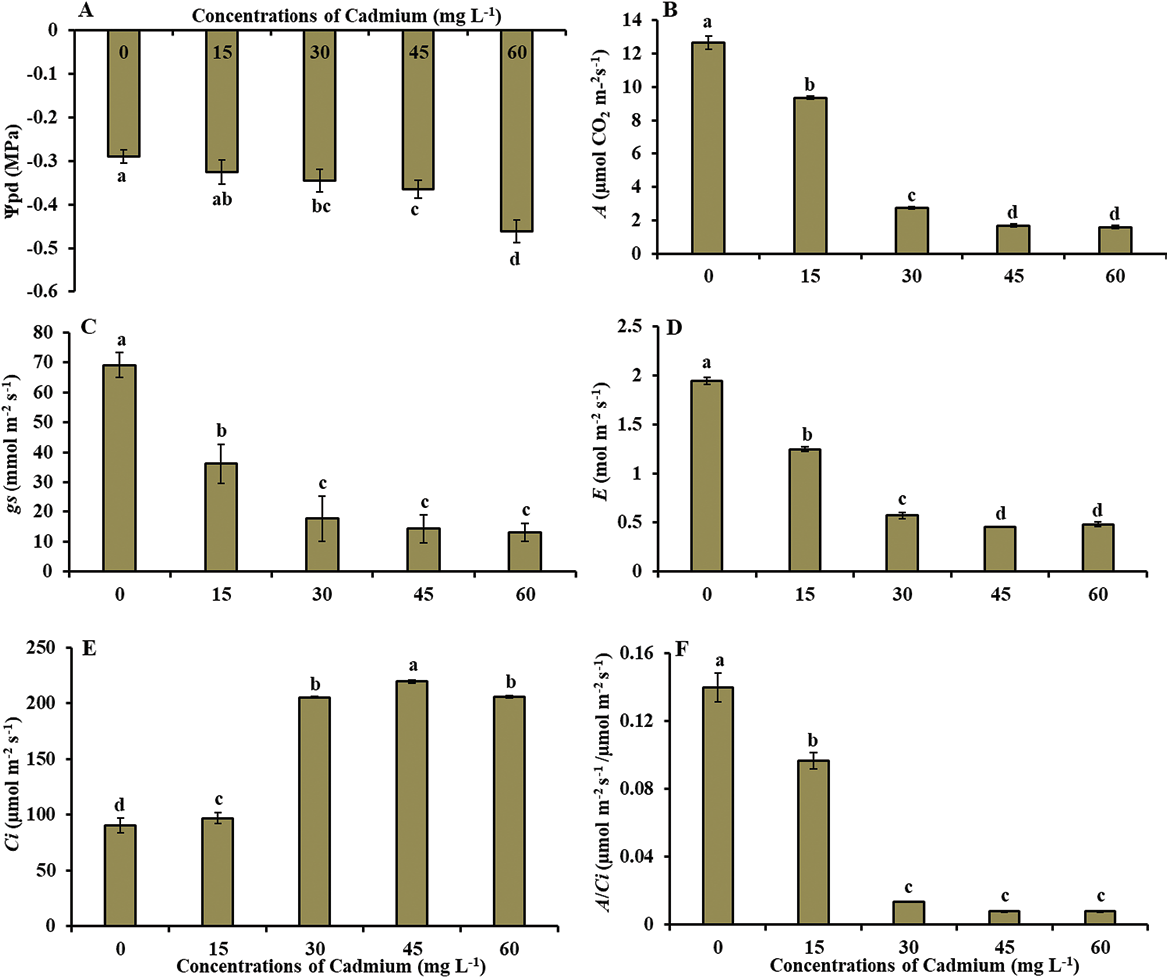
(A) Predawn water potential (Ψ_pd_), (B) net photosynthetic rate (*A*), (C) stomatal conductance (*gs*), (D) transpiration (*E*), (E) internal CO_2_ concentration (*Ci*) and (F) net photosynthesis to intercellular CO_2_ concentration ratio (*A*/*Ci*) in young plants of *V. surinamensis* exposed to five concentrations of cadmium (0, 15, 30, 45 and 60 mg). Different letters for concentrations of cadmium in solution indicate significant differences in the Tukey’s test (*P* < 0.05). Mean ± SD, *n* = 7.

**Figure 2.**
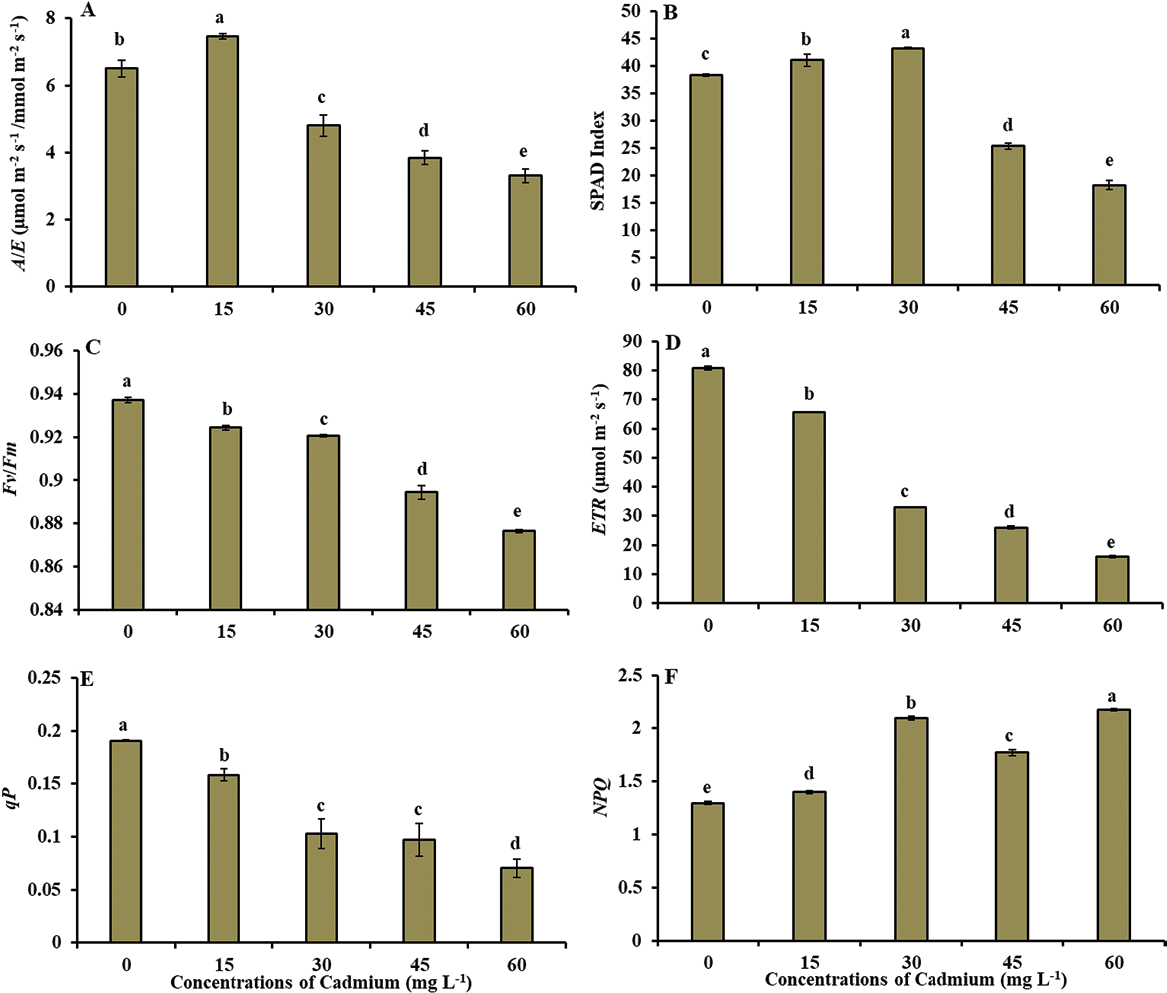
(A) Instantaneous water-use efficiency (*A*/*E*), (B) total chlorophyll (SPAD index), (C) maximum photochemical efficiency of PSII (*Fv*/*Fm*), (D) electron transport rate (*ETR*), (E) photochemical quenching coefficient (*qP*) and (F) non-photochemical quenching coefficient (*NPQ*) in plants young of *V. surinamensis* exposed to five concentrations of cadmium (0, 15, 30, 45 and 60 mg). Different letters for concentrations of cadmium in solution indicate significant differences in the Tukey’s test (*P* < 0.05). Mean ± SD, *n* = 7.

The Ψ_pd_, gas exchange variables (*A*, *gs*, *E*, *Ci*, *A*/*Ci* and *A*/*E*) and total chlorophyll content (SPAD index) were significantly affected by exposure to Cd (Figs 1 and 2; see Supporting Information—Appendix S1). The Ψ_pd_ reduced from −0.29 MPa (control) to −0.46 MPa (concentration of 60 mg L^−1^ of Cd) (Fig. 1A). Lowest values of net CO_2_ assimilation (1.6 μmol m^−2^ s^−1^), stomatal conductance to water vapour (13.0 mmol m^−2^ s^−1^) and transpiration (0.48 mol m^−2^ s^−1^) were obtained in concentration of 60 mg L^−1^ of Cd (Fig. 1B–D). Intercellular CO_2_ concentration increased from 90.6 μmol m^−2^ s^−1^ (control) to 206.0 μmol m^−2^ s^−1^ (concentration of 45 mg L^−1^ of Cd) (Fig. 1E). The *A/Ci* ratio decreased from 0.13 µmol m^−2^ s^−1^/µmol m^−2^ s^−1^ (control) to 0.007 µmol m^−2^ s^−1^/µmol m^−2^ s^−1^ (concentration of 60 mg L^−1^ of Cd) (Fig. 1F). Water-use efficiency (*A/E*) reached the lowest value (3.3 µmol m^−2^ s^−1^/mol m^−2^ s^−1^ in concentration of 60 mg L^−1^ of Cd) (Fig. 2A). Total chlorophyll content (SPAD index) ranged from 38.3 (control) to 18.2 (concentration of 60 mg L^−1^ of Cd) (Fig. 2B).

### Effect of Cd on the fluorescence of chlorophyll *a*

The fluorescence of chlorophyll *a* parameters were significantly affected by exposure to Cd (Fig. 2). *Fv*/*Fm* index decreased from 0.93 (control) to 0.87 (concentration of 60 mg L^−1^ of Cd) (Fig. 2C). *ETR* and *qP* reduced from 80.8 and 0.19 (control) to 15.9 and 0.07 (concentration of 60 mg L^−1^ of Cd), respectively (Fig. 2D and E). *NPQ* increased from 1.3 (control) to 2.17 (concentration of 60 mg L^−1^ of Cd) (Fig. 2F).

### Concentration of Cd in different tissues

**Figure 3.**
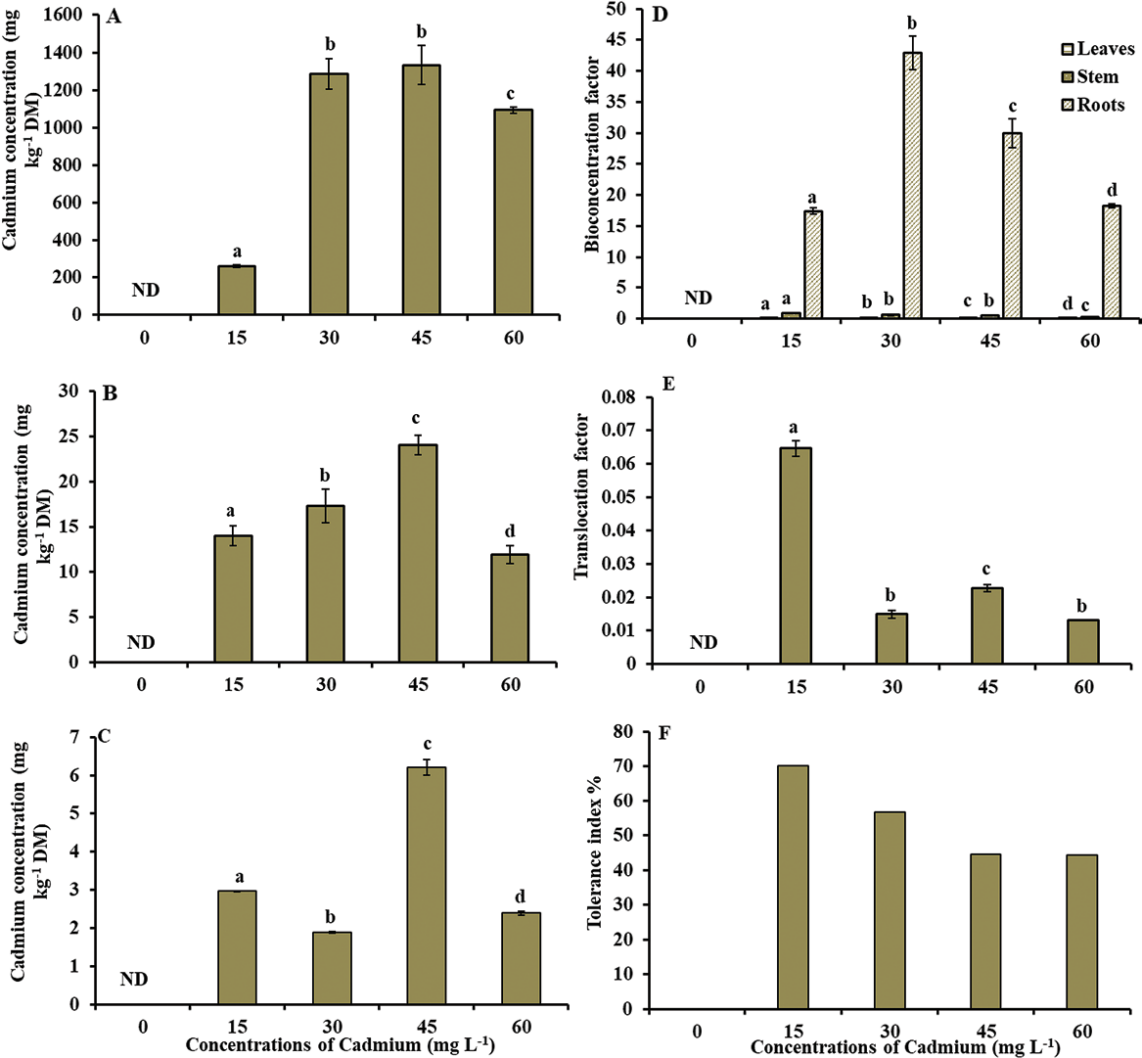
(A) Cadmium concentration in the roots, (B) cadmium concentration in the stem, (C) cadmium concentration in the leaves, (D) bioconcentration factor, (E) translocation factor and (F) tolerance index in young plants of *V. surinamensis* exposed to five concentrations of cadmium (0, 15, 30, 45 and 60 mg). ND = not detected; DM = dry mass. Different letters for concentrations of cadmium in solution indicate significant differences in the Kruskal–Wallis test (*P* < 0.05). Mean ± SD, *n* = 7.

The amount of Cd in the roots and shoot of *V. surinamensis* increased as Cd concentrations increased in the nutrient solution (Fig. 3), being the root system the plant tissue that promoted a higher Cd accumulation, with the highest value of 1333.5 mg kg^−1^ DM at the concentration of 45 mg L^−1^ of Cd (Fig. 3A). In the stem and leaves, the highest values of Cd (23.9 and 6.2 mg kg^−1^ DM, respectively) were obtained at the concentration of 45 mg L^−1^ of Cd (Fig. 3B and C). According to Fig. 3, *V. surinamensis* presented Cd contents in the different plant tissues, as the order root > stem > leaf.

### BCF, TF and TI

In the plants of *V. surinamensis*, BCF was higher at the concentrations of 30 mg L^−1^ of Cd (42.93) and 45 mg L^−1^ of Cd (29.95) (Fig. 3D). The maximum value of TF (0.065) and TI (70.1 %) occurred at the concentration of 15 mg L^−1^ of Cd (Fig. 3E and F; see Supporting Information—Appendix S2).

## Discussion

The data of Ψ_pd_ (Fig. 1A) in young plants of *V. surinamensis* submitted to Cd concentrations indicated that, at a low variation in Ψ_pd_ value, the symptoms of water deficit began, such as the reduction of *gs* (Fig. 1C). The decrease of *gs* (Fig. 1C) in *V. surinamensis* submitted to Cd exposure is probably due to stomatal closure, reduction of stomata density, decrease in pore size (Elloumi *et al.* 2014) and decrease in stomatal size (Di Baccio *et al.* 2014; Nikolić *et al.* 2017). Reduction of stomatal conductance related to water potential was observed in *Eucalyptus camaldulensis* exposed to Cd (Marques *et al*. 2011).

According to Nikolić *et al.* (2017), changes in the stomata promote concomitant limitations in the diffusion of water vapour and CO_2_ into the cells and influence carbon assimilation and loss of the photosynthetic activity of the plant submitted to the presence of Cd. On the other hand, the reduction of *gs* in young plants of *V. surinamensis* may have been a strategy of tolerance to Cd to reduce its absorption and maintain the amount of water in the tissues for plant survival. Reduction of *gs*, *E* and *A* were also observed in other tree species exposed to Cd (Nikolić *et al.* 2017; Pereira *et al*. 2017).

Lowest levels of total chlorophyll in *V. surinamensis* exposed to Cd (Fig. 2B) suggest alteration in chlorophyll biosynthesis or degradation. Cadmium influences chlorophyll biosynthesis because the metal affects water relations (Oláh *et al.* 2015), limits the absorption and transport processes and utilization of Mg^2+^ and Fe^2+^ (He *et al.* 2013; Di Baccio *et al.* 2014; Huang *et al.* 2015) and/or replaces Mg^2+^ in chlorophyll molecules, causing a disturbance of glutathione availability and inhibiting in activity of δ-aminolevulinic dehydratase enzyme (ALA-D) and function of proto-chlorophyll reductase (Parmar *et al.* 2013). Decrease in chlorophyll levels was also observed in other studies with arboreal species submitted to Cd (Yang *et al.* 2015; Nikolić *et al.* 2017).

The reduction in chlorophyll content by increased degradation or decreased biosynthesis may have been reflected in significant reductions in photosynthetic activity of plants under stress by Cd (Elloumi *et al.* 2014; Hernández *et al.* 2015; Yang *et al.* 2015; Michel-López *et al.* 2016; Zouari *et al.* 2016; Nikolić *et al.* 2017; Silva *et al.* 2017).

The reduction of *gs* by limiting the CO_2_ influx in leaves may influence the reduction of photosynthetic rate (Di Bacio *et al.* 2014). Thus, the reduction of *gs* would result in low mesophilic conductance to CO_2_ and consequently in lower chloroplastidic CO_2_, justifying the decrease of photosynthesis in *V. surinamensis* (Fig. 1B). The influence of *gs* on decrease of photosynthesis in plants exposed to Cd was also observed in *C. brasiliense* (Pereira *et al*. 2017). However, the increase in intercellular CO_2_ concentration (*Ci*) (Fig. 1E) with concomitant reductions of *gs* and *A* (Fig. 1C and B) in plants exposed to Cd suggests that decline of photosynthetic activity in *V. surinamensis* also occurs by non-stomatal limitation. The same behaviour was observed in other tree species (Nikolić *et al.* 2017). It has been reported that CO_2_ fixation in chloroplast stroma may be affected by inhibition of enzymes of Calvin cycle (Li *et al.* 2015), such as RuBisCO (Parmar *et al.* 2013; Tran and Popova 2013; Yang *et al.* 2015), contributing to lower *A* values of plant. The inhibition of enzymes related to biochemical stage of photosynthesis, caused by exposure of plants to Cd, may have impaired the fixation and assimilation of photosynthetic CO_2_ and result in increase of *Ci*. In addition, the reduction of instantaneous efficiency of carboxylation (*A*/*Ci*) (Fig. 1F) in plants exposed to Cd reinforces the indication that high concentrations of metal may result in damage to biochemical aspects of photosynthesis causing losses in CO_2_ assimilation rate.

Changes in stomatal opening, water balance and photosynthetic activity are known damages in plants exposed to Cd (Di Bacio *et al.* 2014). In this study, the combined effect of Ψ_pd_ and *gs* reduction on plants exposed to Cd may have been the cause on reduction of *E* (Fig. 1D). The influence of stomatal conductance on transpiration of plants submitted to Cd concentrations was observed in other tree species (Nikolić *et al.* 2017). The reduction of transpiration in plants exposed to Cd can limit the transport of metal from roots to leaves and reduce the damage caused by photosynthetic apparatus (Gratão *et al.* 2015). Thus, the decrease of *E* in *V. surinamensis* in presence of Cd may have been a strategy of tolerance to metal for protection, maintenance or reduction of damages in photosystem components, in an attempt to plant survive.

The reduced values of WUE (*A/E*) (Fig. 2A) in plants submitted to Cd are due to the low photosynthetic rate per unit of water loss in the plants, in which *V. surinamensis* showed a higher sensitivity to higher Cd concentrations. According to Pajević *et al.* (2009), the reduction of WUE in plants exposed to Cd occurs due to the inhibition of the absorption and transport of water, which causes changes in water balance and hence a low production of photoassimilates. Different behaviour was observed in *C. brasiliense* exposed to Cd (Pereira *et al*. 2017).

The effects of Cd stress on chlorophyll fluorescence parameters (*Fv/Fm*, *qP*, *ETR* and *NPQ*) (Fig. 2) may indicate an inhibition of the activity of PSII, resulting in changes in the photosynthetic rate of plants (Pajević *et al.* 2009; Tang *et al.* 2015). The reduction of *Fv/Fm* values (Fig. 2C) in plants exposed to Cd were followed by a reduction of photosynthesis at a carboxylation level, evidenced by an increase in *Ci* (Fig. 1E). The results obtained in present study in relation to chlorophyll fluorescence were evidenced in other tree species (Pietrini *et al.* 2009; Di Baccio *et al*. 2014; Ge *et al.* 2015).

Although the reduction of *A* was followed by a significant decrease in *Fv/Fm* and *ETR* (Fig. 2C and D) in plants submitted to Cd, the effect of the metal may not have been sufficient to cause damage to Φ. This occurs because plants that present *Fv/Fm* values close to 0.85 are considered healthy (Kalaji and Guo 2008; Nikolić *et al.* 2015), i.e. the maximum photochemical quantum efficiency of PSII was not affected by Cd, indicating the stability of thylakoid structure and the efficient flux of electrons through PSII, but with disturbances at a carboxylation level (Pajević *et al.* 2009). According to Nikolić *et al.* (2015), other disturbances, in addition to those in thylakoid and chloroplast membranes, may be involved in the reduction of photosynthesis in plants exposed to heavy metals. On the other hand, significant reductions in *Fv/Fm*, associated with low *qP* values (Fig. 2E), may reduce the photosynthetic efficiency of plants, as suggested by Pietrini *et al.* (2009) for poplar clones and Huang *et al.* (2015) for *Cornus controversa* treated with Cd.

The reduction of *qP* (Fig. 2E) and increase of *NPQ* (Fig. 2F) showed that the damage to PSII induced by a high Cd dose was not enough to cause the photoinactivation, with PSII being protected by an effective dissipation mechanism of heat to avoid the photoinhibition at the reaction centres (Li *et al.* 2015; Tang *et al.* 2015), these findings are corroborated by Ge *et al.* (2015).

Concentrations of 5–10 µg Cd g^−1^ of DM in leaf tissue have been reported to be toxic to most plants (White and Brown 2010). Thus, tolerant plants are often exclusionary, limiting the entry and translocation of heavy metals from the root to shoot (Gallego *et al.* 2012). The high amount of Cd accumulated in the root of *V. surinamensis* (Fig. 3A) indicates the ability to absorb the Cd of the solution and retain the metal especially in the roots, which suggests exclusion and chelation of the metal in the cellular and subcellular compartments of the root system. This may have contributed to a restricted Cd transport from root to the shoot of plants, being a strategy to protect the photosynthetic apparatus, as well as the higher capacity of tolerance of the plant to Cd (Dai *et al.* 2013). The highest concentration of Cd in root was observed in other tree species (Nikolić *et al.* 2017; Pereira *et al*. 2017). Cadmium retention in roots occurs because the metal binds to functional groups, such as thiol, present in the cell wall components of plants (Mehes-Smith *et al.* 2013) and in other compounds such as glutathione (Hasanuzzaman *et al.* 2017), metallothioneins and phytochelatins (Hernandez *et al.* 2015). Some of these compounds were observed in cell wall of root system of plants exposed to Cd (Fernández *et al*. 2014), suggesting that cell wall of *V. surinamensis* root system may have functioned as a barrier to Cd translocation, justifying the higher concentration of metal in root. This is because, at least in part, the lignification can make the cell wall less penetrable, forming a barrier against the Cd influx or even bonding with the metal (Parrota *et al*. 2015).

The phytoextraction capacity can be evaluated by BCF and TF. The BCF evaluates the efficiency of the plant in accumulating metal in relation to the soil solution, while the TF demonstrates the ability of the plant to transport metal from root to shoot (Fan *et al.* 2011). Bioconcentration factor and TF, in aerial tissues of the plant, >1.0 are good indicators of the phytoextraction capacity of Cd (Dai *et al.* 2011). With the exception of Cd hyperaccumulating plants that have BCF and TF > 1 and accumulate >100 mg kg^−1^ DM of Cd in shoot, most plants have BCF < 1.0 (Van der Ent 2013). In this study, the BCF of the aerial part and the TF < 1.0 (Fig. 3D and E) indicate that *V. surinamensis* has low capacity of phytoextraction of Cd and do not belong to the group of hyperaccumulators of this metal. On the other hand, values of BCF and TF < 1 characterize species of metal phytostabilizing plants (Masarovičová *et al.* 2010). These plants accumulate more heavy metals from the substrate in their roots, but restrict their transport and entry into the aerial parts (Malik and Biswas 2012; Hosman *et al.* 2017). In this study, the results of BCF and TF (Fig. 3D and E) indicate the ability of the plant to bioconcentrate the Cd in the root, suggesting that *V. surinamensis* develop mechanisms to accumulate the metal in the root, being able to be effective for phytostabilization Cd. The values of BCF and TF in *V. surinamensis* are in agreement with those obtained in other studies (Michel-López *et al.* 2016; Nikolić *et al.* 2017).

The tolerance of *V. surinamensis* to Cd, estimated by TI, based on the total dry mass of the plants, was similar to the other tree species (Wang *et al*. 2016; Nikolić *et al.* 2017). According to the scheme proposed by Lux *et al.* (2004), in relation to the tolerance index, plants may have high tolerance (TI > 60), medium tolerance (TI between 0.35 and 60) and low tolerance (TI < 0.35). The results obtained in this work in relation to TI (Fig. 3F) indicate that *V. surinamensis* present medium and high tolerance to Cd.

## Conclusion

In this study, we demonstrated that changes in Ψ_pd_, *gs* and *E* in *V. surinamenses* exposed to Cd may have limited the transport of metal from roots to leaves.

The results of chlorophyll and gas exchange fluorescence parameters suggest that decrease of net CO_2_ assimilation in *V. surinamensis* is caused by stomatal limitations and changes in PSII with increasing Cd concentration.

The results of BCF and FT demonstrate low plant efficacy in Cd phytoextraction and suggest that *V. surinamensis* may be promising for Cd phytostabilization purposes.

## Sources of Funding

This study was financed in part by the Coordenação de Aperfeiçoamento de Pessoal de Nível Superior - Brasil (CAPES) - Finance Code 001. This study was financed in part by the Coordination of the Improvement of Higher Education Personnel in Brazil (CAPES), Finance Code 001.

## Conflict of Interest

None declared.

## Contributions by the Authors

W.V.A.J., C.F.O.N., and R.S.O. wrote the manuscript; W.V.A.J., and C.F.O.N. designed the study and analysed the data; B.G.S.F. analysed the data; A.V.C.B. helped in statistical analysis; C.B.A., E.D.C., D.J.P.S., J.S.A.T., and A.S.B. helped perform the experiments.

## Supporting Information

The following additional information is available in the online version of this article—

**Appendix S1.** Data used for leaf water potential, leaf gas exchange, total chlorophyll and fluorescence of chlorophyll *a.*

**Appendix S2.** Data used for bioconcentration factor, translocation factor and tolerance index.

plz022_suppl_Supplementary_Appendix_S1

plz022_suppl_Supplementary_Appendix_S2

## References

[CIT0001] Abdul Qados AMS 2015 Phytoremediation of PB and CD by native tree species grown in the Kingdom of Saudi Arabia. India Journal Scientific Research and Technology 3:22–34.

[CIT0002] Ahmad I, Akhtar MJ, Zahir ZA, Naveed M, Mitter B, Sessitsch A 2014 Cadmium-tolerant bacteria induce metal stress tolerance in cereals. Environmental Science and Pollution Research International 21:11054–11065. 24849374 10.1007/s11356-014-3010-9

[CIT0003] Ali H, Khan E, Sajad MA 2013 Phytoremediation of heavy metals–concepts and applications. Chemosphere 91:869–881. 23466085 10.1016/j.chemosphere.2013.01.075

[CIT0004] Bashir H, Qureshi MI, Ibrahim MM, Iqbal M 2015 Chloroplast and photosystems: impact of cadmium and iron deficiency. Photosynthetica 53:1–15.

[CIT0005] Brasil 2009 Ministério do Meio Ambiente. Resolução Conama n. 420, de 28 de dezembro de 2009. Diário Oficial da República Federativa do Brasil, Brasília 249:81–84.

[CIT0063] Costa BC, Suzuki PM, Martins WBR, Andrade VMS, Oliveira FA 2017 Dinâmica da massa seca e propriedades químicas da liteira em *Virola surinamensis* e floresta sucessional na Amazônia oriental. Revista Verde de Agroecologia e Desenvolvimento Sustentável 12:23–28.

[CIT0006] Dai H, Shan C, Jia G, Lu C, Yang T, Wei A 2013 Cadmium detoxification in *Populus* x *canescens*. Turkish Journal of Botany 37:950–955.

[CIT0007] Dai ZY, Shu WS, Liao B, Wan CY, Li JT 2011 Intraspecific variation in cadmium tolerance and accumulation of a high-biomass tropical tree *Averrhoa carambola* L.: implication for phytoextraction. Journal of Environmental Monitoring 13:1723–1729. 21566812 10.1039/c1em10054h

[CIT0064] Di Baccio D, Castagna A, Tognetti R, Ranieri A, Sebastiani L 2014 Early responses to cadmium of two poplar clones that differ in stress tolerance. Journal Plant Physiology 171:1693–1705. 10.1016/j.jplph.2014.08.007 25213704

[CIT0008] Elloumi N, Zouari M, Chaari L, Jomni C, Rouina BB, Abdallah FB 2014 Ecophysiological responses of almond (*Prunus dulcis*) seedlings to cadmium stress. Biologia 69:604–609.

[CIT0065] Ge W, Jiao Y, Zou J, Jiang W, Liu D 2015 Ultrastructural and photosynthetic response of *Populus* 107 leaves to cadmium stress. Polish Journal of Environmental Studies 24:519–527.

[CIT0009] Fan KC, Hsi HC, Chen CW, Lee HL, Hseu ZY 2011 Cadmium accumulation and tolerance of mahogany (*Swietenia macrophylla*) seedlings for phytoextraction applications. Journal of Environmental Management 92:2818–2822. 21741155 10.1016/j.jenvman.2011.06.032

[CIT0010] Fernández R, Bertrand A, Reis R, Mourato MP, Martins LL, González A 2013 Growth and physiological responses to cadmium stress of two populations of *Dittrichia viscosa* (L.) Greuter. Journal of Hazardous Materials 244:555–562. 23183345 10.1016/j.jhazmat.2012.10.044

[CIT0066] Fernández R, Fernández-Fuego D, Bertrand A, González A 2014 Strategies for Cd accumulation in *Dittrichia viscosa* (L.) Greuter: role of the cell wall, non-protein thiols and organic acids. Plant Physiology and Biochemistry 78:63–70. 24636908 10.1016/j.plaphy.2014.02.021

[CIT0011] Gallego SM, Pena LB, Barcia RA, Azpilicueta CE, Iannone MF, Rosales EP, Zawoznik MS, Groppaa MD, Benavides MP 2012 Unravelling cadmium toxicity and tolerance in plants: insight into regulatory mechanisms. Environmental and Experimental Botany 83:33–46.

[CIT0012] Ge W, Jiao Y, Zou J, Jiang W, Liu D 2015 Ultrastructural and photosynthetic response of *Populus* 107 leaves to cadmium stress. Polish Journal of Environmental Studies 24:519–527.

[CIT0013] Genty B, Briantais JM, Baker NR 1989 The relationship between the quantum yield of photosynthetic electron transport and quenching of chlorophyll fluorescence. Biochimica et Biophysica Acta 990:87–92.

[CIT0014] Gratão PL, Monteiro CC, Tezotto T, Carvalho RF, Alves LR, Peters LP, Azevedo RA 2015 Cadmium stress antioxidant responses and root-to-shoot communication in grafted tomato plants. Biometals 28:803–816. 26077192 10.1007/s10534-015-9867-3

[CIT0067] Hasanuzzaman M, Nahar K, Anee TI, Fujita M 2017 Glutathione in plants: biosynthesis and physiological role in environmental stress tolerance. Physiology Molecular Biology Plants 23:249–268. 10.1007/s12298-017-0422-2 PMC5391355 28461715

[CIT0015] He J, Li H, Luo J, Ma C, Li S, Qu L, Gai Y, Jiang X, Janz D, Polle A, Tyree M, Luo ZB 2013 A transcriptomic network underlies microstructural and physiological responses to cadmium in *Populus* x *canescens*. Plant Physiology 162:424–439. 23530184 10.1104/pp.113.215681 PMC3641221

[CIT0016] Hernández LE, Sobrino-Plata J, Montero-Palmero MB, Carrasco-Gil S, Flores-Cáceres ML, Ortega-Villasante C, Escobar C 2015 Contribution of glutathione to the control of cellular redox homeostasis under toxic metal and metalloid stress. Journal of Experimental Botany 66:2901–2911. 25750419 10.1093/jxb/erv063

[CIT0017] Hosman ME, El-Feky SS, Mohamed MI, Shaker EM 2017 Mechanism of phytoremediation potential of flax (*Linum usitatissimum* L.) to Pb, Cd and Zn. Asian Journal of Plant Science and Research 7:30–40.

[CIT0018] Huang X, Jiang Y, Cheng X, Deng L, Liu X 2015 Photosynthetic performance and anti-oxidative response of *Cornus controversa* seedlings under cadmium and lead stress. Bangladesh Journal of Botany 44:215–221.

[CIT0020] Jesus SV, Marenco RA 2008 O SPAD-502 como alternativa para a determinação dos teores de clorofila em espécies frutíferas. Acta Amazon 38:815–818.

[CIT0021] Kalaji HM, Guo P 2008 Chlorophyll fluorescence: a useful tool in barley plant breeding programs. In: Sánchez A, Gutierrez SJ, eds. Photochemistry research progress. New York: Nova Science Publishers, 12:469–463.

[CIT0022] Khan MA, Khan S, Khan A, Alam M 2017 Soil contamination with cadmium, consequences and remediation using organic amendments. The Science of the Total Environment 601–602:1591–1605. 10.1016/j.scitotenv.2017.06.030 28609847

[CIT0024] Krause GH, Weis E 1991 Chlorophyll fluorescence and photosynthesis: the basics. Annual Review of Plant Physiology and Plant Molecular Biology 42:313–349.

[CIT0025] Li S, Yang W, Yang T, Chen Y, Ni W 2015 Effects of cadmium stress on leaf chlorophyll fluorescence and photosynthesis of *Elsholtzia argyi*–a cadmium accumulating plant. International Journal of Phytoremediation 17:85–92. 25174428 10.1080/15226514.2013.828020

[CIT0026] Lux A, Sottníková A, Opatrná J, Greger M 2004 Differences in structure of adventitious roots in Salix clones with contrasting characteristics of cadmium accumulation and sensitivity. Physiologia Plantarum 120:537–545. 15032815 10.1111/j.0031-9317.2004.0275.x

[CIT0027] Malik N, Biswas AK 2012 Role of higher plants in remediation of metal contaminated sites. Scientific Reviews Chemical Communications 2:141–146.

[CIT0070] Marques TCLLSM, Soares AM, Gomes MP, Martins G 2011 Respostas fisiológicas e anatômicas de plantas jovens de Eucalipto expostas ao cádmio. Revista Árvore 35:997–1006.

[CIT0029] Masarovičová E, KráĬova´ K, Kummerová M 2010 Principles of classification of medicinal plants as hyperaccumulators or excluders. Acta Physiologiae Plantarum 32:823–829.

[CIT0030] Maxwell K, Johnson GN 2000 Chlorophyll fluorescence–a practical guide. Journal of Experimental Botany 51:659–668. 10938857 10.1093/jxb/51.345.659

[CIT0031] Mehes-Smith M, Nkongolo K, Cholewa E 2013 Coping mechanisms of plants to metal contaminated soil. In: Silvern S, ed. Environmental change and sustainability. Vol. 54. Rijeka, Croatia: In Tech, 53–90.

[CIT0032] Michel-López CY, Gil FE, Ortíz GF, Santamaría JM, González-Mendoza D, Ceceña-Duran C, Juarez OG 2016 Bioaccumulation and effect of cadmium in the photosynthetic apparatus of *Prosopis juliflora*. Chemical Speciation & Bioavailability 28:1–4, 1–6.

[CIT0033] Miyazawa M, Pavan MA, Muraoka T, Carmo CAFS, Melo WJ 2009 Chemical analysis of plant tissues. In: Silva FC, ed. Manual of chemical analysis of soils, plants and fertilizers. Brasília, Brazil: Embrapa Informação Tecnológica, 191–233.

[CIT0034] Nikolić NP, Borišev MK, Pajević SP, Arsenov DD, Župunski MD, Orlović SS, Pilipović AR 2015 Photosynthetic response and tolerance of three willow species to cadmium exposure in hydroponic culture. Archives of Biological Sciences 67:1411–1420.

[CIT0035] Nikolić N, Zorić L, Cvetković I, Pajević S, Borišev M, Orlović S, Pilipović A 2017 Assessment of cadmium tolerance and phytoextraction ability in young *Populus deltoides* L. and *Populus* x *euramericana* plants through morpho-anatomical and physiological responses to growth in cadmium enriched soil. Iforest 10:635–644.

[CIT0036] Oláh V, Hepp A, Mészáros I 2015 Comparative study on the sensitivity of turions and active fronds of giant duckweed (*Spirodela polyrhiza* (L.) Schleiden) to heavy metal treatments. Chemosphere 132:40–46. 25777504 10.1016/j.chemosphere.2015.01.050

[CIT0037] Oliveira ES, Oliveira GMTS, Melo NFAC 2017 Concentração de hidrocarbonetos alifáticos e metais pesados na zona portuária de Vila do Conde, Rio Pará-Brasil. Revista Espacios 38:1–12.

[CIT0038] Pajević S, Borisev M, Nikolic N, Krstic B, Pilipović A, Orlović S 2009 Phytoremediation capacity of poplar (*Populus* spp.) and willow (*Salix* spp.) clones in relation to photosynthesis. Archives of Biological Sciences 61:239–247.

[CIT0039] Parmar P, Kumari N, Sharma V 2013 Structural and functional alterations in photosynthetic apparatus of plants under cadmium stress. Botanical Studies 54:45. 28510881 10.1186/1999-3110-54-45 PMC5430381

[CIT0068] Parrotta L, Guerriero G, Sergeant K, Cai G, Hausman JF 2015 Target or barrier? The cell wall of early- and later-diverging plants vs cadmium toxicity: differences in the response mechanisms. Frontiers Plant Science 2:133. 10.3389/fpls.2015.00133 PMC4357295 25814996

[CIT0040] Pietrini F, Zacchini M, Iori V, Pietrosanti L, Ferretti M, Massacci A 2009 Spatial distribution of cadmium in leaves and its impact on photosynthesis: examples of different strategies in willow and poplar clones. Plant Biology 12:355–363. 10.1111/j.1438-8677.2009.00258.x 20398241

[CIT0069] Pereira AS, Cortez PA, Almeida AAF, Prasad MNV, França MGC, Cunha M, Jesus RM, Mangabeira PAO 2017 Morphology, ultrastructure, and element uptake in *Calophyllum brasiliense* Cambess. (*Calophyllaceae* J. Agardh) seedlings under cadmium exposure. Environmental Science Pollution Reseach 24:15576–15588. 10.1007/s11356-017-9187-y 28516356

[CIT0042] Pinheiro HA, Silva JV, Endres L, Ferreira VM, Camara CA, Cabral FF, Oliveira JF, Carvalho LWT, Santos JM, Santos Filho BG 2008 Leaf gas exchange, chloroplastic pigments and dry matter accumulation in castor bean (*Ricinus communis* L.) seedlings subjected to salt stress conditions. Industrial Crops and Products 27:385–392.

[CIT0043] Ramos AM, Santos LAR, Fortes LTG 2009 Normais climatológicas do Brasil 1961-1990, 1a edição. Brasília: INMET.

[CIT0046] Sarruge JR 1975 Soluções nutritivas. Summa Phytopathologica 1:231–233.

[CIT0047] Seyler PT, Boaventura GR 2003 Distribution and partition of trace metals in the Amazon basin. Hydrological Processes 17:1345–1361.

[CIT0048] Sharma S, Rana S, Thakkar A, Baldi A, Murthy RSR, Sharma RK 2016 Physical, chemical and phytoremediation technique for removal of heavy metals. Journal of Heavy Metal Toxicity and Diseases 1:1–15.

[CIT0049] Silva JRR, Fernandes AR, Silva Junior ML, Santos CRC, Lobato AKS 2017 Tolerance mechanisms in *Cassia alata* exposed to cadmium toxicity – potential use for phytoremediation. Photosynthetica 55:1–10.

[CIT0050] Silvestre WVD, Silva PA, Palheta LF, Oliveira Neto CF, Souza R, Festucci-Buselli RA, Pinheiro HA 2017 Differential tolerance to water deficit in two açaí (*Euterpe oleracea* Mart.) plant materials. Acta Physiologiae Plantarum 39:1–10.

[CIT0051] Solti Á, Sárvári É, Szöllősi E, Tóth B, Mészáros I, Fodor F, Szigeti Z 2016 Stress hardening under long-term cadmium treatment is correlated with the activation of antioxidative defence and iron acquisition of chloroplasts in *Populus*. Zeitschrift Fur Naturforschung. C, Journal of Biosciences 71:323–334. 27542199 10.1515/znc-2016-0092

[CIT0052] Song Y, Jin L, Wang X 2016 Cadmium absorption and transportation pathways in plants. International Journal of Phytoremediation 19:133–141. 10.1080/15226514.2016.1207598 27409403

[CIT0053] Statistical Analysis System Institute – SAS 2007 SAS® 9.1.3 (TS1M3) for Windows Microsoft. Cary, NC: SAS Institute.

[CIT0054] Tang Y, Bao Q, Tian G, Fu K, Cheng H 2015 Heavy metal cadmium tolerance on the growth characteristics of industrial hemp (*Cannabis sativa* L.) in China. In: Chen S, Zhou S, eds. International Conference on Advances in Energy, Environment and Chemical Engineering. Amsterdam: Atlantis Press, 289–295.

[CIT0055] Tran TA, Popova LP 2013 Functions and toxicity of cadmium in plants: recent advances and future prospects. Turkish Journal of Botany 37:1–13.

[CIT0056] Van der Ent A, Baker AJM, Reeves RD, Pollard AJ, Schat H 2013 Hyperaccumulators of metal and metalloid trace elements: facts and fiction. Plant Soil 362:319–334.

[CIT0071] Wang Y, Gu C, Bai S, Sun Z, Zhu T, Zhu X, Grit DH, Tembrock LR 2016 Cadmium accumulation and tolerance of Lagerstroemia indica and Lagerstroemia fauriei (Lythracaeae) seedlings for phytoremediation applications. International Journal of Phytoremediation 18:1104–1112. 27196684 10.1080/15226514.2016.1183581

[CIT0057] White PJ, Brown PH 2010 Plant nutrition for sustainable development and global health. Annals of Botany 105:1073–1080. 20430785 10.1093/aob/mcq085 PMC2887071

[CIT0058] Wilkins DA 1957 A technique for the measurement of lead tolerance in plants. Nature 180:37–38.13451634

[CIT0060] Yang Y, Li X, Yang S, Zhou Y, Dong C, Ren J, Sun X, Yang Y 2015 Comparative physiological and proteomic analysis reveals the leaf response to cadmium-induced stress in poplar (*Populus yunnanensis*). PLoS One 10:1–20. 10.1371/journal.pone.0137396 PMC4562643 26349064

[CIT0061] Zhao S, Shen Z, Duo L 2015 Heavy metal uptake and leaching from polluted soil using permeable barrier in DTPA-assisted phytoextraction. Environmental Science and Pollution Research International 22:5263–5270. 25354438 10.1007/s11356-014-3751-5

[CIT0062] Zouari M, Ben Ahmed C, Elloumi N, Bellassoued K, Delmail D, Labrousse P, Ben Abdallah F, Ben Rouina B 2016 Impact of proline application on cadmium accumulation, mineral nutrition and enzymatic antioxidant defense system of *Olea europaea* L. cv Chemlali exposed to cadmium stress. Ecotoxicology and Environmental Safety 128:195–205. 26946284 10.1016/j.ecoenv.2016.02.024

