# Supplementary material for: Effect of cadmium on young plants of Virola surinamensis
Source: AoB Plants. 2019 Apr 5;11(3):plz022. doi: 10.1093/aobpla/plz022 (PMC6524489; doi:10.1093/aobpla/plz022)
Supplement: plz022_suppl_Supplementary_Appendix_S1 [file plz022_suppl_supplementary_appendix_s1.pdf]

TREATMENT	SPAD	A	gs_mmol	Ci	E	A_E	A_Ci	FV_FM	NPQ	ETR	qP	Ψpd
0 mg Cd	38,33	12,30	71,48	82,77	1,95	6,31	0,15	0,94	1,28	81,61	0,19	-0,29
0 mg Cd	38,15	12,30	71,48	98,33	1,88	6,55	0,13	0,94	1,28	80,79	0,19	-0,28
0 mg Cd	38,80	12,39	62,84	87,60	1,98	6,26	0,14	0,94	1,29	79,97	0,19	-0,31
0 mg Cd	38,20	12,39	73,09	82,77	1,98	6,26	0,15	0,94	1,31	79,97	0,19	-0,31
0 mg Cd	38,15	13,30	73,09	98,33	1,95	6,82	0,14	0,94	1,31	81,61	0,19	-0,29
0 mg Cd	38,47	12,80	67,16	90,55	1,96	6,51	0,14	0,94	1,30	81,20	0,19	-0,28
0 mg Cd	38,40	13,05	65,00	94,44	1,92	6,79	0,14	0,94	1,29	80,58	0,19	-0,27
15 mg Cd	42,28	9,33	39,94	101,43	1,25	7,48	0,09	0,92	1,39	65,53	0,16	-0,30
15 mg Cd	41,90	9,43	25,51	91,90	1,28	7,40	0,10	0,92	1,40	65,33	0,16	-0,35
15 mg Cd	41,90	9,23	42,01	101,43	1,22	7,58	0,09	0,92	1,42	65,33	0,15	-0,37
15 mg Cd	41,20	9,43	42,01	100,95	1,28	7,40	0,09	0,93	1,41	65,72	0,15	-0,30
15 mg Cd	41,30	9,23	39,94	89,86	1,22	7,58	0,10	0,92	1,38	65,72	0,17	-0,34
15 mg Cd	39,85	9,38	33,76	95,64	1,26	7,44	0,10	0,92	1,39	65,62	0,16	-0,30
15 mg Cd	39,12	9,41	29,63	98,30	1,27	7,42	0,10	0,93	1,39	65,67	0,16	-0,32
30 mg Cd	43,44	2,73	16,20	204,91	0,57	4,83	0,01	0,92	2,10	32,72	0,11	-0,32
30 mg Cd	43,06	2,66	26,65	205,83	0,63	4,24	0,01	0,92	2,07	33,10	0,13	-0,37
30 mg Cd	43,10	2,66	16,20	205,37	0,57	4,71	0,01	0,92	2,09	32,72	0,11	-0,30
30 mg Cd	43,30	2,72	27,41	204,91	0,54	5,02	0,01	0,92	2,09	32,91	0,10	-0,35
30 mg Cd	43,30	2,84	6,54	205,83	0,54	5,23	0,01	0,92	2,12	33,10	0,08	-0,36
30 mg Cd	43,20	2,78	11,37	205,60	0,60	4,67	0,01	0,92	2,09	32,81	0,10	-0,36
30 mg Cd	43,25	2,81	19,01	205,26	0,57	4,93	0,01	0,92	2,11	32,86	0,09	-0,36
45 mg Cd	25,50	1,65	19,45	221,42	0,45	3,63	0,01	0,89	1,75	26,21	0,08	-0,35
45 mg Cd	24,50	1,65	10,37	217,62	0,45	3,63	0,01	0,90	1,81	26,90	0,10	-0,33
45 mg Cd	25,50	1,69	19,45	219,52	0,46	3,70	0,01	0,89	1,77	26,05	0,12	-0,37
45 mg Cd	25,50	1,87	15,97	217,62	0,46	4,09	0,01	0,89	1,81	25,54	0,10	-0,38
45 mg Cd	26,50	1,59	7,30	221,42	0,45	4,05	0,01	0,89	1,74	25,54	0,11	-0,39
45 mg Cd	25,00	1,73	11,63	218,57	0,46	3,79	0,01	0,89	1,77	26,22	0,08	-0,38
45 mg Cd	25,25	1,71	15,54	220,00	0,45	4,03	0,01	0,90	1,75	25,88	0,08	-0,36
60 mg Cd	18,10	1,48	13,17	204,52	0,48	3,10	0,01	0,88	2,18	16,23	0,08	-0,42
60 mg Cd	17,30	1,74	13,17	204,52	0,48	3,65	0,01	0,88	2,16	15,20	0,07	-0,45
60 mg Cd	17,30	1,71	17,24	206,85	0,51	3,34	0,01	0,88	2,17	16,09	0,08	-0,45
60 mg Cd	19,70	1,48	9,42	206,85	0,46	3,24	0,01	0,88	2,17	16,09	0,07	-0,46
60 mg Cd	18,10	1,60	16,93	205,69	0,46	3,51	0,01	0,88	2,18	16,23	0,06	-0,47
60 mg Cd	18,90	1,61	11,29	206,27	0,49	3,26	0,01	0,88	2,18	15,71	0,07	-0,48
60 mg Cd	18,50	1,54	10,35	206,56	0,50	3,07	0,01	0,88	2,18	15,97	0,06	-0,50
