# Supplementary material for: Effect of cadmium on young plants of Virola surinamensis
Source: AoB Plants. 2019 Apr 5;11(3):plz022. doi: 10.1093/aobpla/plz022 (PMC6524489; doi:10.1093/aobpla/plz022)
Supplement: plz022_suppl_Supplementary_Appendix_S2 [file plz022_suppl_supplementary_appendix_s2.pdf]

TREATMENT		BCF_Root	BCF_Stem	BCF_Leaf	TF	TI	Cd_Root
0 mg Cd	T1R1						
0 mg Cd	T1R2						
0 mg Cd	T1R3						
0 mg Cd	T1R4						
0 mg Cd	T1R5						
0 mg Cd	T1R6						
0 mg Cd	T1R7						
15 mg Cd	T2R1	17,86163	0,990406667	0,193566667	0,066285838	71,493321	267,9245
15 mg Cd	T2R2	17,86163	0,990406667	0,193566667	0,066285838	69,255548	267,9245
15 mg Cd	T2R3	17,86163	0,990406667	0,193566667	0,066285838	69,071669	267,9245
15 mg Cd	T2R4	17,86163	0,990406667	0,193566667	0,066285838	70,037273	267,9245
15 mg Cd	T2R5	16,84961	0,854126667	0,19552	0,062295	68,152174	252,7442
15 mg Cd	T1R6	16,84961	0,854126667	0,19552	0,062295	72,726157	252,7442
15 mg Cd	T1R7	16,84961	0,854126667	0,19552	0,062295	70,666667	252,7442
30 mg Cd	T3R1	45,09787	0,52812	0,06354	0,013119468	58,062977	1352,936
30 mg Cd	T3R2	45,09787	0,52812	0,06354	0,013119468	56,299213	1352,936
30 mg Cd	T3R3	45,09787	0,52812	0,06354	0,013119468	59,64406	1352,936
30 mg Cd	T2R4	45,09787	0,52812	0,06354	0,013119468	57,039798	1352,936
30 mg Cd	T3R5	40,04203	0,64158	0,062373333	0,017580359	55,797101	1201,261
30 mg Cd	T1R6	40,04203	0,64158	0,062373333	0,017580359	57,002578	1201,261
30 mg Cd	T1R7	40,04203	0,64158	0,062373333	0,017580359	52,916667	1201,261
45 mg Cd	T4R1	31,48936	0,552624444	0,134682222	0,021826635	44,391102	1417,021
45 mg Cd	T4R2	31,48936	0,552624444	0,134682222	0,021826635	44,544858	1417,021
45 mg Cd	T4R3	31,48936	0,552624444	0,134682222	0,021826635	44,125782	1417,021
45 mg Cd	T4R4	31,48936	0,552624444	0,134682222	0,021826635	43,344956	1417,021
45 mg Cd	T4R5	27,15893	0,507528889	0,14312	0,023957086	43,88285	1222,152
45 mg Cd	T1R6	27,15893	0,507528889	0,14312	0,023957086	46,360624	1222,152
45 mg Cd	T1R7	27,15893	0,507528889	0,14312	0,023957086	44,803571	1222,152
60 mg Cd	T5R1	18,467	0,210733333	0,045806667	0,013891807	44,513359	1108,02
60 mg Cd	T5R2	18,467	0,210733333	0,045806667	0,013891807	45,430685	1108,02
60 mg Cd	T5R3	18,467	0,210733333	0,045806667	0,013891807	42,087542	1108,02
60 mg Cd	T5R4	18,467	0,210733333	0,045806667	0,013891807	44,078394	1108,02
60 mg Cd	T5R5	17,92059	0,180378333	0,031776667	0,011838618	44,299517	1075,235
60 mg Cd	T1R6	17,92059	0,180378333	0,031776667	0,011838618	45,906469	1075,235
60 mg Cd	T1R7	17,92059	0,180378333	0,031776667	0,011838618	44,642857	1075,235

Cd_Stem	Cd_Leaf
14,8561	2,9035
14,8561	2,9035
14,8561	2,9035
14,8561	2,9035
12,8119	2,9328
12,8119	2,9328
12,8119	2,9328
15,8436	1,9062
15,8436	1,9062
15,8436	1,9062
15,8436	1,9062
19,2474	1,8712
19,2474	1,8712
19,2474	1,8712
24,8681	6,0607
24,8681	6,0607
24,8681	6,0607
24,8681	6,0607
22,8388	6,4404
22,8388	6,4404
22,8388	6,4404
12,644	2,432725
12,644	2,432725
12,644	2,432725
12,644	2,432725
10,8227	2,3275
10,8227	2,3275
10,8227	2,3275
